# Nationwide usage of ethambutol and incidence and screening practices of optic neuropathy

**DOI:** 10.3389/fphar.2024.1461111

**Published:** 2024-10-15

**Authors:** Jiyeong Kim, Hyeon Yoon Kwon, Seong Joon Ahn

**Affiliations:** ^1^ Department of Pre-Medicine, College of Medicine and Biostatistics Laboratory, Medical Research Collaborating Center, Hanyang University, Seoul, Republic of Korea; ^2^ Department of Ophthalmology, Hanyang University Hospital, Hanyang University College of Medicine, Seoul, Republic of Korea

**Keywords:** ethambutol, toxic optic neuropathy, incidence, practice patterns, screening

## Abstract

**Introduction:**

Ethambutol, a key anti-tuberculosis medication, is associated with optic neuropathy, which can lead to significant vision impairment. This population-based cohort study investigated the nationwide usage of ethambutol and the incidence and screening of optic neuropathy among ethambutol users.

**Methods:**

The cohort included 206,157 individuals from the Health Insurance Review and Assessment database in South Korea who initiated ethambutol therapy between 1 January 2015, and 31 December 2021. Among them, 117,309 individuals without prior ophthalmic diseases were analyzed to investigate practice patterns. The timing of the baseline examinations (an ophthalmic examination immediately preceding [within 1 month] ethambutol initiation or the first ophthalmic assessment conducted after the initiation) and subsequent monitoring and modalities used were assessed.

**Results:**

The cumulative incidences of the optic neuropathy categories, overall optic neuropathy, optic neuropathy/optic neuritis, and optic atrophy, were analyzed. The annual number of ethambutol users declined over time between 2015 and 2021. The cumulative incidences of overall optic neuropathy, optic neuropathy/optic neuritis, and optic atrophy were 2.8%, 2.0%, and 0.7%, respectively. Optical coherence tomography usage for ophthalmic examinations remarkably increased from 23.9% in 2015 to 85.5% in 2021, while visual field examinations were frequently (69.6%) employed for functional assessment at the time of diagnosis. Baseline screening within 6 months of initiating ethambutol therapy was performed in 12.0%–13.1% of ethambutol initiators annually over the study period. The percentage of ethambutol initiators receiving subsequent monitoring within 6 months following baseline screening declined over time, from 13.1% in 2015 to 7.9% in 2021.

**Discussion:**

The diminishing trend in monitoring emphasizes the need for improved pharmacovigilance to reduce the risk of optic atrophy and blindness through early detection.

## 1 Introduction

Ethambutol, an antibiotic used against *Mycobacterium* infections, is an effective treatment against tuberculosis. However, ethambutol use often leads to a serious ocular side effect, ethambutol-induced optic neuropathy (EON) (Chen et al., 2015; Chamberlain et al., 2017; MacVinish et al., 2024), which potentially leads to bilateral optic atrophy and vision loss (MacVinish et al., 2024). The exact mechanism of EON is unclear, although it is speculated to involve either reduced copper availability and oxidative phosphorylation in human mitochondria or inhibited lysosomal activation due to zinc chelation (Kozak et al., 1998; Chung et al., 2009; Huang et al., 2015).

EON affects approximately 1%–2% of patients undergoing treatment for *Mycobacterium tuberculosis*, potentially impacting approximately 100,000 individuals worldwide annually (Lee et al., 2008; Chen et al., 2012; Ezer et al., 2013; Chen et al., 2015). The risk of EON correlates significantly with the dosage of ethambutol administered, with doses below 15 mg/kg/day carrying a risk of less than 1% and higher doses escalating the risk (Yang et al., 2016). Accordingly, World Health Organization (WHO) guidelines recommend initiating ethambutol treatment at 15–20 mg/kg/day for tuberculosis patients, World Health Organization (2008) but dosing for non-tubercular mycobacterial infections, like *Mycobacterium avium* complex and *M. kansasii*, may vary within a range of 15–25 mg/kg as part of a multidrug regimen (WHO, 2020; Calcagno et al., 2024).

Proactive screening for EON is crucial in preventing vision loss, yet the impact of EON on visual impairment or incidence of blindness remains inadequately studied. Furthermore, educating patients about the potential of visual loss and the need for regular screening and follow-up with an ophthalmologist is also important (Saxena et al., 2021). The screening should encompass visual acuity, visual field (VF) testing, color vision testing, and dilated fundus examination; thereafter, high-risk patients are recommended to undergo frequent follow-up examinations (Chamberlain et al., 2017). However, despite the potential for irreversible bilateral vision loss, evidence supporting the best practice for screening methods is limited (MacVinish et al., 2024). Furthermore, national-level studies on EON screening practices, ethambutol user trends, and risk factors for EON remain limited, despite prior investigations into associated comorbidities (Chen et al., 2012; Yang et al., 2016; Mandal et al., 2021).

Therefore, this study aimed to investigate the nationwide usage of ethambutol in South Korea, along with the incidence and screening practices of EON, with a focus on understanding the annual trend. By analyzing trend data and real-world screening practices, we aimed to address the areas to be improved for more effective strategies for screening this potentially blinding condition.

## 2 Methods

### 2.1 Study population

**FIGURE 1:**
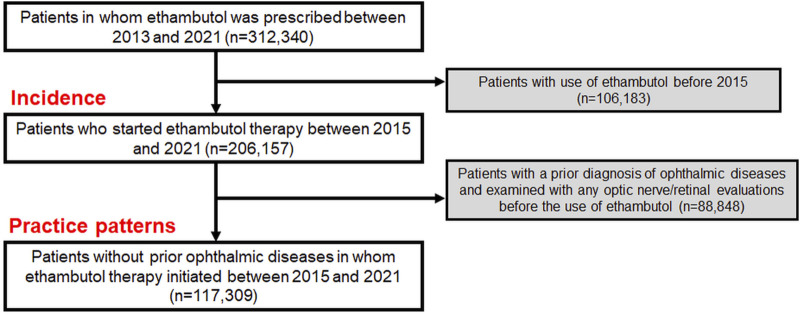
Flowchart indicating the inclusion/exclusion criteria and the number of subjects meeting the criteria.

The cohort for this study included patients from the Health Insurance Review and Assessment database in South Korea, which contains extensive health claim data of approximately 50 million people. This database includes detailed information on medication, medical visits, and demographics, and diagnoses based on the Korean Standard Classification of Diseases (KCD), 7^th^ or 8^th^ Revision, slightly modified from the International Statistical Classification of Diseases and Related Health Problems, Tenth Revision (ICD-10). We identified the individuals who were prescribed ethambutol between 1 January 2013, and 31 December 2021, and excluded those who had received it before 1 January 2015, to finally select those starting the therapy between 2015 and 2021. This exclusion, based on our assumption that individuals who did not use the drug between 2013 and 2015 but started using it after 2015 are ethambutol initiators after 2015, allowed for precise calculation of the duration and cumulative dose of the drug, mirroring methodologies employed in prior studies (Kim et al., 2023; Kim et al., 2024). This resulted in the creation of an overall user group, which we used to analyze the incidence of optic neuropathy (Figure 1). From this population, we excluded individuals who had previously undergone optic nerve/retinal evaluations and were diagnosed with ophthalmic disease before ethambutol use to avoid the inclusion of existing eye condition monitoring and to analyze the practice patterns for screening (Figure 1). Individuals with multiple sclerosis were also excluded to eliminate the possibility of optic nerve disease attributable to this condition. Finally, our study included 117,309 ethambutol users without prior ophthalmic disease, for whom ethambutol therapy was initiated between 1 January 2015, and 31 December 2021. All data recorded in the database between 1 January 2015, and 31 December 2022, were analyzed to monitor screening practices. The inclusion/exclusion criteria and patient numbers are graphically outlined in Figure 1 and the specific procedure and diagnosis codes used in this study are detailed in Supplementary Table S1. This study was approved by the Institutional Review Board (IRB) of Hanyang University Hospital (IRB File no. 2023–11–008) and adhered to the Declaration of Helsinki. Informed consent was waived by the Institutional Review Board of Hanyang University Hospital due to the retrospective nature of the study and the use of deidentified data. The reporting adhered to the Strengthening the Reporting of Observational Studies in Epidemiology (STROBE) guidelines.

### 2.2 Definitions and evaluations

Baseline examination was defined as an ophthalmic examination immediately preceding (within 1 month) ethambutol therapy initiation or the first ophthalmic assessment conducted after the initiation. Subsequent monitoring denoted examinations performed after the baseline assessment. The identification and categorization of optic neuropathy were performed using specific diagnostic codes within the dataset. Overall optic neuropathy was characterized by the presence of optic neuropathy/optic neuritis (H46), optic atrophy (H47.2), or unspecified disorder of the visual pathways (H47.7). We analyzed the categories of optic neuropathy/optic neuritis and optic atrophy separately. Furthermore, we analyzed functional (visual) impairment using the diagnostic codes H54.0 (visual impairment) and 54.4 (blindness, including bilateral blindness). When the first registration of the above diagnostic codes in the database was after the date of ethambutol therapy initiation, we considered it in the EON incidence category.

The screening practices were evaluated using various parameters and outcome measures. Initially, the annual number of overall and new ethambutol users from 2015 to 2021 was evaluated to understand the trends among users. The timing of examinations—baseline or monitoring—along with the time intervals between the commencement of ethambutol use and baseline examination, and between baseline and subsequent monitoring examinations were analyzed. The proportion of patients undergoing baseline screening within 6 months of starting ethambutol therapy and the percentage of patients receiving subsequent monitoring within 3 and 6 months of baseline screening were also analyzed. The techniques employed for screening examinations, such as fundoscopy/fundus photography, optical coherence tomography (OCT), automated VF testing, color vision testing, and visual evoked potentials (VEPs), were documented to identify the preferred screening or diagnostic methods for EON. Finally, the frequency of monitoring examinations per year was calculated.

### 2.3 Statistical analysis

Descriptive statistics were used to summarize the demographic and clinical characteristics of the ethambutol users. Categorical variables are expressed as frequencies and percentages, whereas continuous variables are presented as mean (standard deviation) or median (interquartile range) values. The cumulative incidences of several categories of optic neuropathies were evaluated using Kaplan–Meier curves. All *p*-values were derived from two-sided tests, and statistical significance was set at P < 0.05. Statistical analyses were performed using SAS Enterprise Guide version 7.1 (SAS Institute, Cary, NC).

## 3 Results

### 3.1 Demographic and clinical characteristics

Table 1 provides a comprehensive overview of the demographic and clinical characteristics of the ethambutol users included in this study. Among the 206,157 users, the majority were male (57.9%). The mean age of users was 60.2 ± 19.0 years, with a notable distribution across different age groups. The most common systemic diseases among the users included diabetes mellitus and hypertension, whereas the primary indication for ethambutol use was pulmonary tuberculosis, accounting for 78.0% of the patients.

**TABLE 1:** Demographic and clinical information of ethambutol users in the present study (n = 206,157).

Characteristics	Mean or value
Sex	
Male	119,324 (57.9%)
Female	86,833 (42.1%)
Mean age (±SD), years	60.2 ± 19.0
<30	17,594 (8.5%)
30–39	16,502 (8.0%)
40–49	23,549 (11.4%)
50–59	35,909 (17.4%)
60–69	35,494 (17.2%)
≥70	77,109 (37.4%)
Systemic diseases	
Diabetes mellitus	72,119 (35.0%)
Hypertension	93,785 (45.5%)
Liver diseases	97,036 (47.1%)
Kidney diseases	38,815 (18.8%)
Dementia	4,846 (2.4%)
Rheumatoid arthritis	10,936 (5.3%)
Indication for ethambutol use	
Pulmonary tuberculosis	160,725 (78.0%)
Extra-pulmonary tuberculosis	22,888 (11.1%)
Others (e.g. miliary tuberculosis)	22,544 (10.9%)
Mean duration of ethambutol use (±SD), months	12.1 ± 11.7
Less than 3 months	43,083 (20.9%)
3–6 months	27,526 (13.4%)
6 months–1 year	44,360 (21.5%)
1–1.5 years	46,874 (22.7%)
1.5–2 years	22,325 (10.8%)
≥2 years	21,989 (10.7%)
Mean daily dose of ethambutol (±SD), mg/day	902.9 ± 193.7
<750 mg	11,376 (5.5%)
750–1,000 mg	138,550 (67.2%)
1,000–1,250 mg	52,251 (25.4%)
≥1,250 mg	3,980 (1.9%)
Mean cumulative dose of ethambutol (±SD), g	315.4 ± 301.2
<200 g	84,038 (40.8%)
200–400 g	60,435 (29.3%)
400–600 g	36,426 (17.7%)
≥600 g	25,258 (12.3%)

SD, standard deviation.

Table 1 also presents detailed information regarding the duration of ethambutol use, mean daily dose, and cumulative dose among the study participants. The mean duration of ethambutol use was 12.1 ± 11.7 months, showing varying proportions across different duration categories, with 20.9%, 13.4%, 21.5%, 22.7%, 10.8%, and 10.7% of users having used it for <3 months, 3–6 months, 6 months to 1 year, 1–1.5 years, 1.5–2 years, and ≥2 years, respectively. Additionally, the mean daily dose of ethambutol revealed that 5.5%, 67.2%, 25.4%, and 1.9% of users were prescribed <750, 750–<1,000, 1,000–<1,250, and ≥1,250 mg/day, respectively. Additionally, the cumulative dose of ethambutol, which reflects the total amount of the drug taken over the course of treatment, was 315.4 ± 301.2 g on average.

### 3.2 Ethambutol users and their trend over time

Table 2 illustrates the usage trends of ethambutol in South Korea, including the annual number of patients using and initiating ethambutol therapy between 2015 and 2021. In 2015, 56,139 patients used ethambutol and 36,863 individuals initiated ethambutol therapy, accounting for 0.110% and 0.072% of the Korean population, respectively. The subsequent years saw a gradual decline in the total number of users and annual number of individuals starting ethambutol therapy. For instance, by 2021, 34,316 patients used ethambutol and 21,512 initiated use, representing approximately 0.066% and 0.042% of the population, respectively.

**TABLE 2:** Annual number of patients using ethambutol and those who initiated ethambutol therapy between 2015 and 2021.

Year	Total number of patients using ethambutol (% among entire Korean population in each year^a^)	Annual number of patients who initiated ethambutol therapy (% among Korean population^a^)
2015	56,139 (0.110%)	36,863 (0.072%)
2016	52,297 (0.102%)	34,559 (0.067%)
2017	48,971 (0.095%)	32,268 (0.063%)
2018	46,011 (0.089%)	30,156 (0.058%)
2019	42,731 (0.083%)	27,636 (0.053%)
2020	37,414 (0.072%)	23,167 (0.045%)
2021	34,316 (0.066%)	21,512 (0.042%)

^a^ Obtained by dividing the number of ethambutol users by that of the entire Korean population in each year (from 51,014,947 in 2015 to 51,744,876 in 2021)

### 3.3 Incidences of several optic neuropathy conditions and the modalities used at the time of diagnosis

**FIGURE 2:**
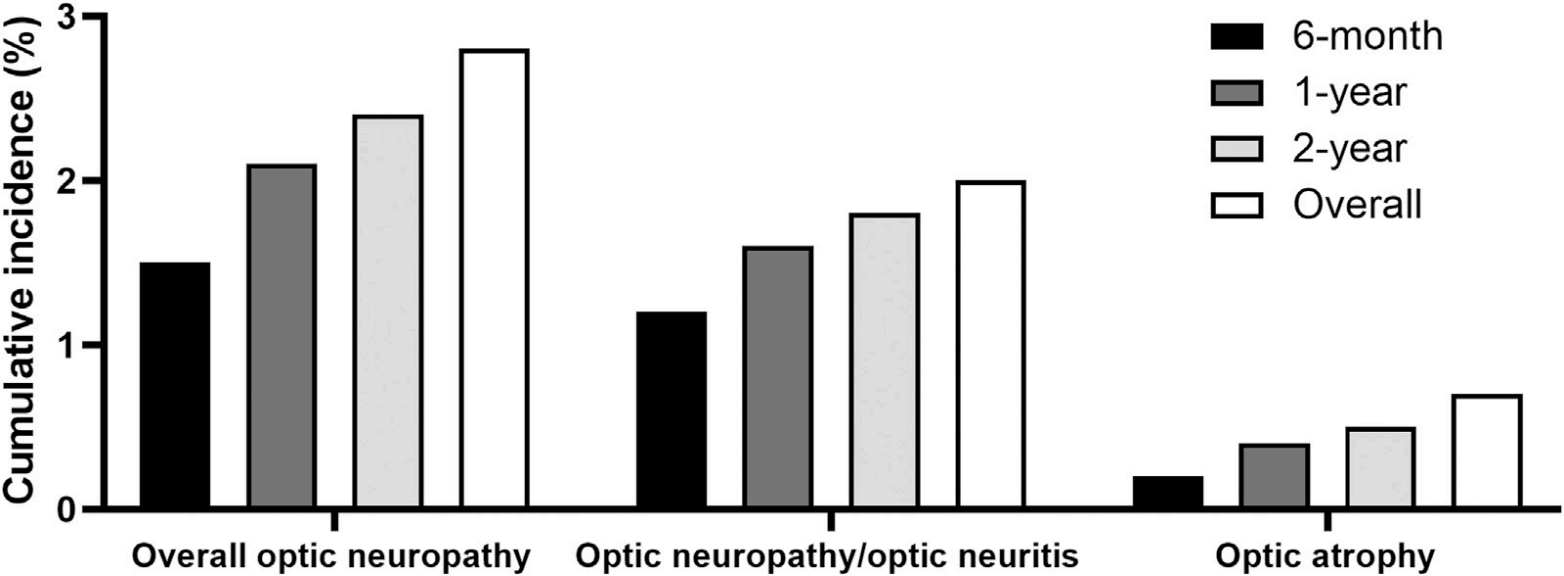
Cumulative incidences of overall optic neuropathy, optic neuropathy/optic neuritis, and optic atrophy among ethambutol users.

Figure 2 illustrates the cumulative incidences of optic neuropathies, including overall optic neuropathy, optic neuropathy/optic neuritis, and optic atrophy, among ethambutol users during different time periods between 2015 and 2021. The cumulative incidence of overall optic neuropathy, optic neuropathy/optic neuritis, and optic atrophy during the entire study period was 2.8%, 2.0%, and 0.7%, respectively. One-year cumulative incidences for these conditions were 2.1%, 1.6%, and 0.4%, respectively. Supplementary Table S2 presents the onset (time at detection) of overall optic neuropathy, which was less than 6 months in more than half of the cases.

**FIGURE 3:**
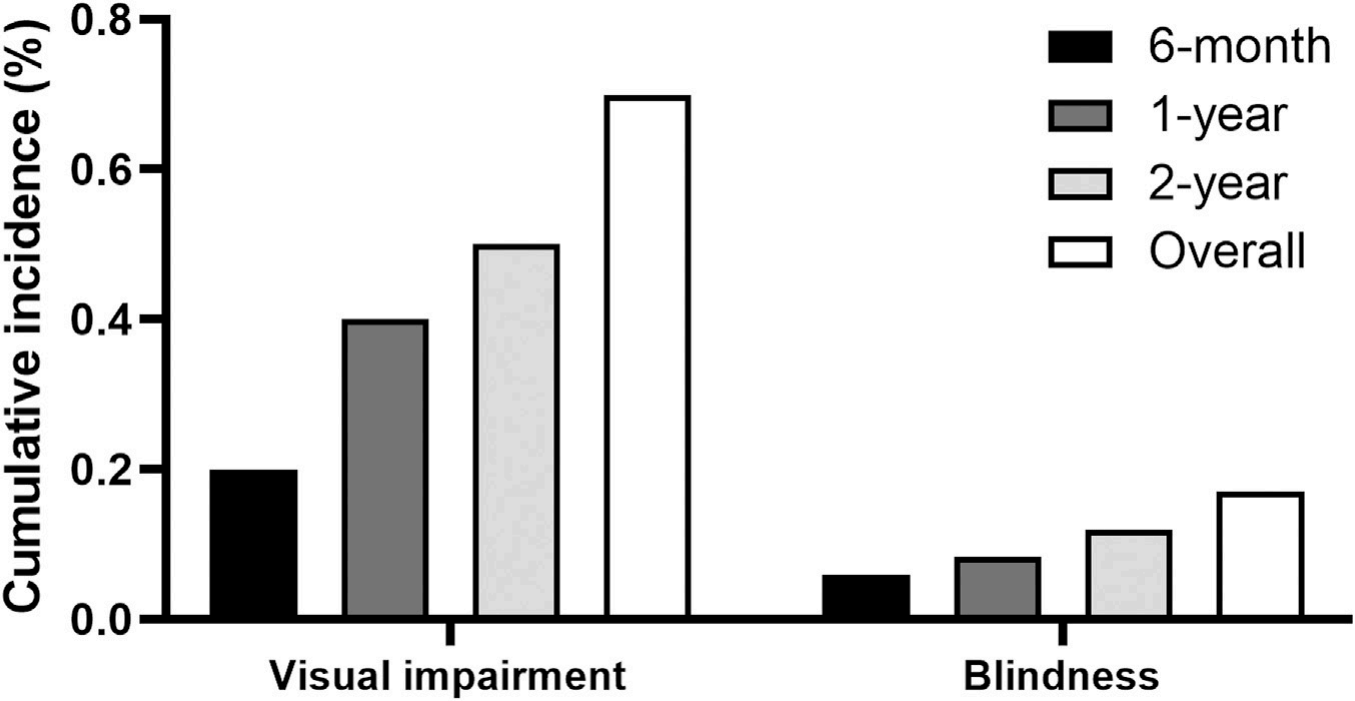
Cumulative incidences of visual impairment and blindness among ethambutol users.

Figure 3 displays the cumulative incidences of visual impairment and blindness among individuals receiving ethambutol. The data demonstrates the progression of visual impairment over time, offering an overview of the functional impairment associated with ethambutol use. The 1-year cumulative incidences of visual impairment and blindness were 0.4% and 0.08%, respectively, whereas the 2-year incidence increased to 0.5% and 0.1%, respectively.

Supplementary Table S3 outlines the tests performed at the time of diagnosis among the patients diagnosed with optic neuropathy. Fundoscopy/fundus photography was most commonly used at the time of diagnosis as 88.4% received the test, followed by automated VFs (69.6%), OCT (63.8%), color vision testing (35.5%), VEPs (14.6%), and others (including pattern or multifocal electroretinogram; 2.9%).

### 3.4 Screening practice patterns of ethambutol optic neuropathy

Table 3 provides the descriptive statistics regarding the timing and modalities used for baseline examinations and subsequent monitoring among ethambutol users between 2015 and 2021. Among the total number of users, approximately 31.9% received any ophthalmic examination after initiating ethambutol therapy, with a notable percentage undergoing examinations within specific time frames, such as within 3 months (9.2%), 6 months (12.5%), and 1 year (16.5%) post-ethambutol use. The median timing for baseline examination since ethambutol use, indicating the typical duration before initial ophthalmic evaluation, was 336 days in the screened population. Additionally, approximately 56.1% of the patients who underwent baseline screening received subsequent monitoring examinations, with an average of 0.42 monitoring examinations per year. The mean timing for the first monitoring examination since baseline examination was approximately 10.0 ± 15.4 months.

**TABLE 3:** Descriptive statistics of the timing and modalities used for the baseline examination (ophthalmic examination immediately preceding [within 1 month] to ethambutol therapy initiation or first examination after ethambutol use) and monitoring (follow-up screening) among all patients between 2015 and 2021.

Characteristics	Value
Timing	
No. of patients receiving baseline examinations/total number of ethambutol users (%)	37,469/117,309 (31.9%)
No. of patients receiving ophthalmic examination within 3 months of ethambutol use/No. of users (%)	10,742/117,309 (9.2%)
No. of patients receiving ophthalmic examination within 6 months of ethambutol use/No. of users (%)	14,645/117,309 (12.5%)
No. of patients receiving ophthalmic examination within 1 year of ethambutol use/No. of users (%)	19,335/117,309 (16.5%)
Timing of the baseline examination since ethambutol use, median (Q1-Q3), days	336 (67–1,001)
No. of patients receiving any monitoring examinations after baseline screening/No. of patients receiving baseline screening (%)	21,031/37,469 (56.1%)
No. of monitoring examinations per year after baseline screening, numbers/year	0.42 ± 0.46
Mean/median (Q1–Q3) timing of the 1^st^ monitoring examination since baseline examination, months	10.0 ± 15.4/2.7 (0.6–12.7)
Mean/median (Q1–Q3) interval of monitoring between 1^st^ and 2^nd^ monitoring exam, months	6.6 ± 11.4/1.9 (0.4–6.9)
Mean/median (Q1–Q3) interval of monitoring between 2^nd^ and 3^rd^ monitoring exam, months	5.6 ± 9.9/1.6 (0.4–6.1)
Modalities used for screening	Baseline/Monitoring (%)
Funduscopy/fundus photography	36,257 (96.8%)/20,382 (96.9%)
Optical coherence tomography	10,901 (29.1%)/6,675 (31.7%)
Automated visual fields	8,038 (21.5%)/4,901 (23.3%)
Color vision test	3,235 (8.6%)/2,101 (10.0%)
Visual evoked potentials	633 (1.7%)/393 (1.9%)
Others^a^	367 (1.0%)/281 (1.3%)

^a^ Others include pattern electroretinography, multifocal electroretinography, and additional modalities.

**FIGURE 4:**
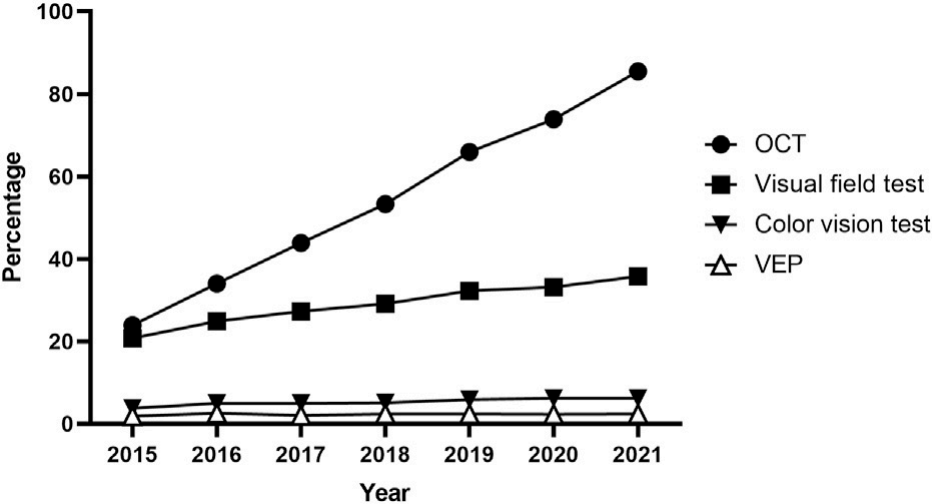
Annual trends in the percentage of patients undergoing optical coherence tomography, visual field testing, visual evoked potentials, and color vision testing for screening among all ethambutol users each year from 2015 to 2021.

Furthermore, Table 3 outlines the modalities used for screening, such as fundoscopy/fundus photography, OCT, automated VF testing, color vision testing, VEPs, and other modalities, including optic disc and optic nerve fiber layer analysis. Fundoscopy/fundus photography was the most frequently employed modality for baseline (96.8%) and monitoring (96.9%) examinations, followed by OCT (29.1% baseline, 31.7% monitoring) and automated VF testing (21.5%, 23.3%). Color vision testing was performed in 3,235 (8.6%) and 2,101 (10.0%) patients for baseline screening and monitoring, respectively. The annual trends in the proportion of patients undergoing OCT, automated VF testing, color vision testing, and VEPs among all ethambutol users each year are presented in Figure 4, indicating a remarkable increase in OCT use and a modest increase in visual fields over time. In contrast, no significant trends were observed in the frequencies of VEP or color vision tests over time.

Table 4 presents the annual trends in the proportion of ethambutol initiators undergoing retinopathy screening, including baseline examination and subsequent monitoring. The data illustrate the percentage of ethambutol users examined within 6 months of initiating therapy for baseline examination among those who initiated ethambutol therapy each year, as well as those with subsequent examinations within a 3- and 6-month time frame from the baseline examination. Notable variations were observed in screening rates over the years, with fluctuating percentages observed for baseline and monitoring examinations. For instance, the proportion of ethambutol initiators undergoing baseline examinations within 6 months decreased from 12.8% in 2015 to 12.0% in 2017, with a slight increase to 13.1% in 2021. Monitoring examination rates varied, with 13.1% examined within 6 months among ethambutol initiators in 2015, followed by a decreasing trend over the years, indicating a downward trend in timely monitoring.

**TABLE 4:** Yearly trends of the proportion of ethambutol users undergoing retinopathy screening (baseline examination and subsequent monitoring).

Year	Baseline examination within 6 months of ethambutol initiation	Monitoring	
		Examined within 3 months of baseline exam	Examined within 6 months of baseline exam
2015	3,358 (12.8%)	2,830 (10.8%)	3,435 (13.1%)
2016	2,819 (12.6%)	2,408 (10.8%)	2,926 (13.1%)
2017	2,287 (12.0%)	1,875 (9.8%)	2,281 (11.9%)
2018	2,021 (12.4%)	1,430 (8.8%)	1,732 (10.6%)
2019	1,684 (12.4%)	1,074 (7.9%)	1,308 (9.6%)
2020	1,304 (12.3%)	687 (6.5%)	851 (8.0%)
2021	1,172 (13.1%)	585 (6.5%)	704 (7.9%)

## 4 Discussion

The present study unveils a nationwide pattern in the utilization of ethambutol and screening practices for optic neuropathy among ethambutol users in South Korea. Our findings indicate a notable reduction in the total number of ethambutol users over time. However, it is concerning that a substantial proportion of ethambutol users did not undergo baseline screening, and the rates of timely monitoring examinations have declined over the study period.

Analysis of the usage trends of ethambutol revealed a significant decrease in the annual number of ethambutol users in South Korea, declining from 56,139 to 34,316, as well as in those initiating ethambutol therapy, decreasing from 36,863 to 21,512 between 2015 and 2021. Despite the decline in numbers, this study emphasizes the substantial population of ethambutol users, exceeding 30,000 individuals annually in South Korea. The decline in ethambutol usage observed in this study may be attributed to a sustained reduction in tuberculosis incidence across the country, resulting in fewer new ethambutol users over time (Cho, 2018). Understanding these trends is vital for evaluating the potential impact of ethambutol on optic neuropathy, both from a public health perspective, particularly in pharmacovigilance, and from an ophthalmological standpoint. Consequently, our findings may be potentially valuable for refining screening strategies to align with the evolving patterns of ethambutol utilization.

The study evaluated the cumulative incidences of several categories of optic neuropathies and modalities used for diagnostic purposes among ethambutol users. As the diagnostic code for EON was missing in our database, several diagnostic codes were employed to identify EON, including overall optic neuropathy, optic neuropathy/optic neuritis, and optic atrophy, enabling a systematic assessment of the potential conditions while maximizing the detection of the ethambutol-induced optic neuropathy. Overall optic neuropathy and optic neuropathy/optic neuritis were noted in 2.8% and 2.0% of the patients, respectively, which are compatible with the estimates of 1–2% among the treated patients, identified in previous studies (Lee et al., 2008; Ezer et al., 2013; Chen et al., 2015; Yang et al., 2016; Chamberlain et al., 2017; Mandal et al., 2021). In our study, optic neuropathy/optic neuritis appeared to be the most specific classification for EON, as a greater proportion of users within this category discontinued the drug after diagnosis compared to overall optic neuropathy cases. However, optic atrophy, a category not previously addressed in studies, was rare (0.7%) in our cohort. This structural finding aligns with the low percentage of vision impairment (0.7%) depicted in Figure 3, although it should not be overlooked. This rarity may be partly attributed to symptom severity prompting patients to seek ophthalmologic care and receive an EON diagnosis, a condition widely recognized among clinicians, before reaching an advanced stage. Otherwise, timely detection and management strategies, such as dose reduction or discontinuation through toxicity screening, could have contributed to the low incidence of optic atrophy.

Funduscopy/fundus photography is the most commonly utilized modality at the time of diagnosis of optic neuropathy, but it has limitations in detecting abnormalities, especially in the early stages of EON. Visual field examinations, reported to be more sensitive than visual acuity, color vision, or contrast sensitivity tests for early detection of ethambutol toxicity (Mandal et al., 2021), were commonly utilized for diagnosis in our cohort (Supplementary Table S3). As OCT has the capability to detect subclinical changes, such as thinning of the peripapillary retinal nerve fiber layer in the retinal nerve fiber layer and ganglion cell layer (Zoumalan et al., 2005; Kim and Hwang, 2009), before the clinical signs of EON become apparent, it may be increasingly favored for screening, a trend supported by our data (Figure 4). However, using OCT for detecting subclinical EON has produced mixed results, with some studies showing increased retinal nerve fiber layer thickness in asymptomatic patients on ethambutol (Zoumalan et al., 2005; Yoo et al., 2020). Although our data indicated a trend toward an objective, structural modality for EON screening, a multimodal approach using structural and functional (e.g. visual acuity, color vision, VFs) evaluations may be desirable for the comprehensive screening of optic neuropathy in patients receiving ethambutol therapy.

This study also provides crucial insights into the current screening practices for EON, highlighting variations in monitoring rates from 2015 to 2021 (Tables 3 and 4). Our findings indicate that only approximately one-third of ethambutol users received any ophthalmic examination after initiating ethambutol therapy. The unscreened population is particularly of concern as this led to late diagnosis at symptomatic or even advanced stages. Furthermore, baseline screening within 6 months of initiating ethambutol therapy was performed in only 12.5% of patients, which is inadequate considering the reported timing of optic neuropathy in previous studies, which can occur as early as 1 month after initiation (Chamberlain et al., 2017). Additionally, the percentage of patients undergoing monitoring within 6 months following baseline screening remained consistently below 15%, declining to only 7.9% in 2021. Further monitoring may seem unnecessary after short-term use of ethambutol and drug discontinuation, which might have contributed to the low proportion of patients receiving monitoring in our study. However, this downward trend is also concerning due to the ongoing necessity for pharmacovigilance among ethambutol users and the risk of blindness associated with EON. Unlike other drugs with known ocular toxicity, such as hydroxychloroquine and pentosan polysulfate, where improved awareness has led to better screening practices (Kim et al., 2023; Kim et al., 2024), EON screening remains insufficiently emphasized. These findings emphasize the critical need for increased vigilance concerning regular, timely screening to detect and manage this vision-threatening condition early and, more importantly, prescribing physicians should prioritize referring patients receiving ethambutol therapy to ophthalmologists for initial screening examinations. Therefore, our study underscores the importance of increasing clinician awareness and establishing standardized screening guidelines, as the development of widely recognized screening protocols will be helpful in reversing this concerning trend.

Despite the valuable insights provided, this study had certain limitations that warrant consideration. The retrospective nature of the study design and reliance on health claim databases may have introduced biases and limitations inherent to national claims data sources (Kumar et al., 2023). Moreover, the characteristics of the study population, all Koreans, may restrict the generalizability of our findings to other populations with distinct healthcare systems and epidemiological profiles on tuberculosis. Furthermore, the use of diagnostic codes for case identification may have led to a misclassification or over- or underreporting of optic neuropathy cases, potentially affecting the accuracy of incidence estimates. Particularly, KCD codes do not include the ICD-10 code H46.3, which is specific for toxic optic neuropathy. Therefore, the inclusion of several optic neuropathy categories, utilized for identification of cases in this study, might lack specificity. Additionally, the distinction between optic neuropathy/neuritis and optic atrophy is somewhat arbitrary and of limited clinical significance. However, we aimed to capture cases of unrecognized or chronic ethambutol optic neuropathy, given the lack of specific information on ethambutol exposure available to screening physicians. Moreover, this study also could not eliminate the possibility of participants undergoing ophthalmic exams for reasons other than toxicity screening (Kim et al., 2023). However, although these tests were conducted for other purposes, they may reveal structural or functional abnormalities of the optic nerve or visual pathways, which may lead to the diagnosis of one of the several categories of optic neuropathy included in this study.

In conclusion, this study shed light on the nationwide trend in the number of ethambutol users at risk of EON and its incidence in South Korea. By elucidating the real-world screening practice patterns, this study offers valuable insights for optimizing screening practices for ethambutol users. Enhancing vigilance for timely and more widely performed screening practices may mitigate the burden of ethambutol-associated optic neuropathy through early detection and management.

## Data Availability

The datasets presented in this article are not readily available because of confidentiality concerns. Requests to access the datasets should be directed to the Health Insurance Review and Assessment (HIRA).
