# Supplementary material for: Nationwide usage of ethambutol and incidence and screening practices of optic neuropathy
Source: Front Pharmacol. 2024 Oct 15;15:1461111. doi: 10.3389/fphar.2024.1461111 (PMC11518835; doi:10.3389/fphar.2024.1461111)
Supplement: Supplementary file 1 [file Table1.DOCX]

**Supplementary Table 1.** Procedure, diagnosis, and drug codes used in the HIRA database

| **Category** | **Definition** | **Codes used** |
| --- | --- | --- |
| **Diagnosis** | Pulmonary tuberculosis | A15, A16 |
|  | Extrapulmonary tuberculosis | A17, A18 |
|  | Diabetes mellitus | E10, E11, E12, E13, E14 |
|  | Hypertension | I10, I11, I12, I13, I15 |
|  | Liver diseases | K70-77 |
|  | Kidney diseases | N00-29 |
|  | Dementia | G30 |
|  | Rheumatoid arthritis | M05, M06 |
|  | Overall optic neuropathy | H46, H47.2, H47.7 |
|  | Optic neuropathy/optic neuritis | H46 |
|  | Optic atrophy | H47.2 |
|  | Visual impairment | H54.0 |
|  | Blindness | H54.4 |
| **Procedure** | Funduscopy/fundus photography | E6660, E6670, E6674 |
|  | Optical coherence tomography | EZ796 |
|  | Automated visual fields | E6690, E6691 |
|  | Color vision test | E6770 |
|  | Visual evoked potentials | FA181, FA182 |
|  | Others | E6685, E6686, E6687, EX798, EZ794 |
| **Drug** | Ethambutol | 155601ATB (200 mg), 155602ATB (400 mg), 155605ATB (800 mg) |
|  | Isoniazid | 178101ATB, 178102ATB |

**Supplementary Table 2**. Onset (time of detection) of optic neuropathy/neuritis

| **Period** | **Number of patients (%)** |
| --- | --- |
| Less than 6 months | 3048 (53.3%) |
| 6 – 12 months | 1259 (22.0%) |
| 12 – 18 months | 415 (7.3%) |
| 18 months or later | 991 (17.4%) |

**Supplementary Table 3.** Tests performed at the time of diagnosis

| **Modalities used** | **Frequency (%)** |
| --- | --- |
| Funduscopy/fundus photography | 1,699 (88.4%) |
| Optical coherence tomography | 1,225 (63.8%) |
| Automated visual fields | 1,337 (69.6%) |
| Color vision test | 681 (35.5%) |
| Visual evoked potentials | 280 (14.6%) |
| Others | 55 (2.9%) |
